# Toluene oxidation by non-thermal plasma combined with palladium catalysts

**DOI:** 10.3389/fchem.2013.00007

**Published:** 2013-06-20

**Authors:** Monica Magureanu, Daniela Dobrin, Nicolae B. Mandache, Bogdan Cojocaru, Vasile I. Parvulescu

**Affiliations:** ^1^Department for Plasma Physics and Nuclear Fusion, National Institute for Lasers, Plasma and Radiation PhysicsMagurele, Bucharest, Romania; ^2^Department of Organic Chemistry, Biochemistry and Catalysis, University of BucharestBucharest, Romania

**Keywords:** non-thermal plasma, Pd/Al_2_O_3_ catalyst, total oxidation of toluene, volatile organic compounds, CO_2_ selectivity

## Abstract

The oxidation of toluene in air was investigated using a dielectric barrier discharge (DBD) combined with a Pd/Al_2_O_3_ catalyst. When using only plasma, rather low selectivity toward CO_2_ was obtained: 32–35%. By filling the DBD reactor with Pd/Al_2_O_3_ catalyst the CO_2_ selectivity was significantly enhanced (80–90%), however, a large amount of toluene was desorbed from the catalyst when the discharge was operated. By filling a quarter of the discharge gap with catalyst and placing the rest of the catalyst downstream of the plasma reactor, an important increase of CO_2_ selectivity (~75%) and a 15% increase in toluene conversion were achieved as compared to the results with plasma alone. The catalyst exhibited a very good stability in this reaction.

## Introduction

The emission of volatile organic compounds (VOC) from transportation and various industrial processes represents a significant source of air pollution, and therefore VOC removal from waste gas with high efficiency and low costs is an issue of major importance for human health and the environment. Non-thermal plasma is highly efficient in producing oxidizing species (such as atomic oxygen, ozone, hydroxyl radicals, etc), which may react with the VOC molecules and decompose them, and therefore various electrical discharges have been investigated for this purpose (Leys and Morent, 2012; Magureanu, 2012). On this basis, in the last years various non-thermal plasmas, such as microwave (Horikoshi et al., 2007), corona (Van Durme et al., 2009) dielectric barrier discharge (DBD), gliding arc (Yang et al., 2009), glow discharge (Zhang et al., 2012) have been widely investigated for the total oxidation of hydrocarbons in air.

However, plasma activation is rather non-selective, so in order to obtain simultaneously high conversion and high selectivity toward total oxidation, the combination of plasma and catalysis appears more promising (Delagrange et al., 2006; Van Durme et al., 2008; Magureanu et al., 2011). Noble metal catalysts (Pd, Pt, Au, Ag) in combination with plasma were tested in many works, due to their high efficiency for VOCs abatement (Harling et al., 2007; Kim et al., 2008; Van Durme et al., 2009). In particular, palladium-based catalysts are well known to be among the most active catalysts for the catalytic total oxidation of methane (Zhu et al., 2004; Huang et al., 2008).

Experiments carried out using plasma also confirmed the superiority of palladium (Yamamoto et al., 2009; Kroker et al., 2012). Loading of palladium is a very important parameter and recent studies demonstrated that concentrations around 2wt% provided the most effective catalysts (Da Costa et al., 2008).

In this work the total oxidation of toluene in air by plasma-assisted catalysis was investigated at room temperature and atmospheric pressure. Non-thermal plasma was generated in a DBD. A palladium catalyst supported on γ-Al_2_O_3_ was used in the experiments. Compared to previous reports this study followed a different route in the preparation of the catalyst trying to answer additional questions like: how susceptible is the palladium catalyst to the type of the precursor and to the textural properties of alumina. For this purpose, this study considered PdCl_2_ as precursor and alumina prepared via a route close to that reported by Da Costa et al. (2008) but with a different texture. The catalysts were placed (i) inside the plasma region (in-plasma configuration), (ii) downstream of the plasma reactor (post-plasma configuration), and (iii) both inside and after the plasma reactor (combined configuration). The effect of applied voltage, or implicitly of the discharge power, was investigated. The results obtained with plasma alone were compared with those achieved in the plasma-catalytic systems.

## Materials and methods

### Materials

The catalysts were prepared by incipient wetness impregnation of γ-Al_2_O_3_ with an acid solution of PdCl_2_ (ReagentPlus®, 99%, from Sigma-Aldrich) then dried after a program of temperatures at 110°C, calcined at 500°C for 2 h and reduced in hydrogen flow at 400°C for 4 h (Pârvulescu et al., 1994). Palladium concentration in the catalysts was 2%.

### Experimental set-up and procedures

The plasma reactor consisted in a quartz tube with the inner diameter of 9 mm. The inner electrode was a metallic rod of 3 mm diameter placed on the axis of the DBD reactor. The outer electrode was aluminum tape placed on the outside of the tube on a length of 48 mm.

The discharge was operated in ac mode, with sinusoidal voltage, at 50 Hz frequency. The electrical circuit is described in detail in (Magureanu et al., 2011). The high voltage was applied to the inner electrode, while the outer electrode was grounded. The discharge voltage was measured by a high voltage probe (Tektronix P6015). The discharge current was determined from the voltage drop across a shunt resistor (Rs = 3 Ω) connected in series with the earthed electrode. The total charge dissipated in the discharge was measured with a non-inductive capacitor (C = 1 μF), placed instead of the shunt resistor. The discharge voltage, current and total charge were monitored by a digital oscilloscope (Tektronix TDS 2022). The amplitude of the applied voltage was varied in the range 10–20 kV. The experimental data were scaled to the specific input energy (SIE), defined as the ratio of the power and flow rate. Ambient air was used as working gas, the total gas flow rate was 163 mL/min and the initial toluene concentration was 50 ppm.

The concentrations of CO_2_ and CO resulting from toluene oxidation were monitored continuously by a gas analyzer (Ultramat 6, Siemens) coupled on-line. Hydrocarbons and oxidation byproducts were analyzed on a HP 5890 Series II Gas Chromatograph equipped with a TCD detector. Prior analysis, reactants and by-products were separated on a Porapaq Q column. The concentration of ozone in the effluent gas was measured by an ozone analyzer (Ozomat MP, Anseros).

### Characterization measurements

The catalysts were characterized using elemental analysis, differential thermal and thermogravimetric analysis, adsorption–desorption isotherms at −196°C, XRD, DRIFTs, and TEM. The differential thermal and thermogravimetric analysis was carried out using a TG-DTA analyzer (Shimadzu DTG-60 Simultaneous DTA-TG Apparatus) on 4–6 mg samples in nitrogen atmosphere under the heating rate of 10°C/min from room temperature to 900°C and using alumina as reference. The C, N, and S content of the samples was determined using a EuroVector Euro EA Elemental Analyzer (combustion elemental analyzer). X-Ray diffraction patterns were recorded using a Shimadzu XRD-7000 diffractometer with Cu *K*α(λ = 1.5418 Å, 40 kV, 40 mA) at a step of 0.02° and a scan rate of 2°/min in the 2θ range of 5–90°. Crystalline phase were identified by comparasion of the XRD patterns with the JCPDS database. FTIR spectra were recorded at room temperature in nitrogen atmosphere with a Thermo Electron Nicolet 4700 spectrometer using a diffuse reflectance (DR) accessory (Smart Collector for DRIFT spectra). The final spectra correspond to an average of 100 scans with 4 cm^−1^ resolution. TEM analysis characterizations were carried out using a JEOL JEM-1010 instrument operating at 100 kV and equipped with a CCD camera and a Tecnai G^2^F20(FI) instrument, respectively.

## Results

### Discharge characteristics

**Figure 1:**
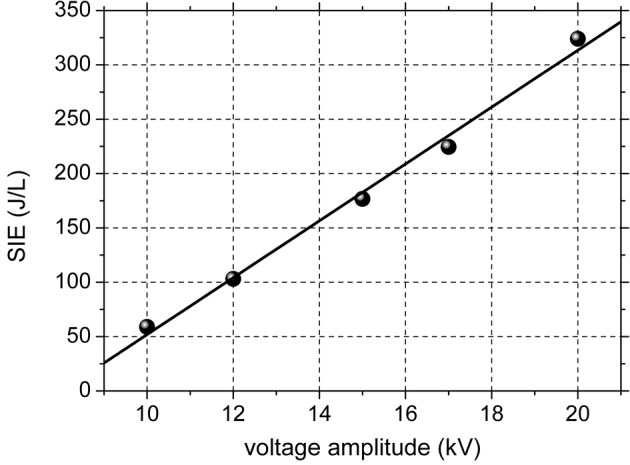
**Specific input energy as a function of amplitude of the discharge voltage**.

The discharge has a filamentary nature, typical for DBDs at atmospheric pressure, consisting in a multitude of plasma filaments of short duration (tens of ns). As previously mentioned, the voltage is sinusoidal and the current consists of several peaks with amplitudes of tens to hundreds of mA and durations of tens of nanoseconds on each alternance of the voltage. The electrical power dissipated in the discharge was calculated by the Lissajous method (Falkenstein and Coogan, 1997). Figure 1 shows the SIE as a function of the amplitude of the applied voltage. The SIE depends approximately linearly on the voltage. When increasing the applied voltage from 10 to 20 kV, the average power increased from 0.16 to 0.88 W and the SIE varies in the range 59–324 J/L. Since the power is very low, thermal effect can be excluded. The formation of nitrogen oxides is also avoided at these low values of discharge power.

### Total oxidation of toluene

**Figure 2:**
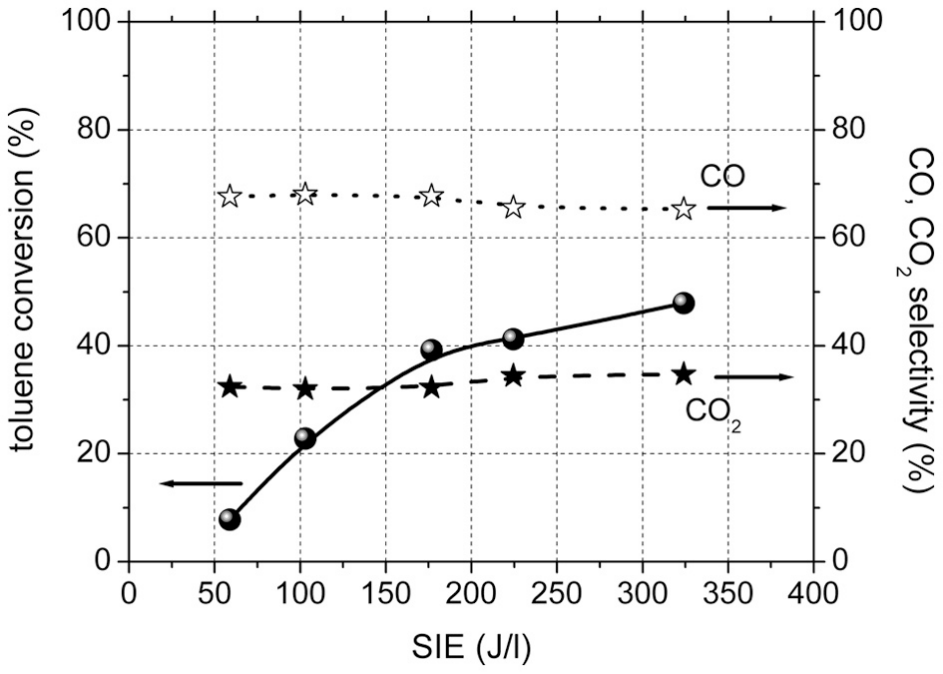
**Toluene conversion and CO and CO2 selectivity in DBD in the absence of catalysts**.

Figure 2 shows toluene conversion and the selectivity to carbon dioxide and carbon monoxide as a function of SIE for the experiments performed in plasma, in the absence of catalysts. The conversion of toluene in plasma was 8% for the lowest input energy used and reached 48% for highest SIE. The only gaseous reaction products of toluene oxidation in the plasma were CO and CO_2_. The selectivity to CO_2_ showed a very slight increase with SIE and ranged between 32 and 35%.

**Figure 3:**
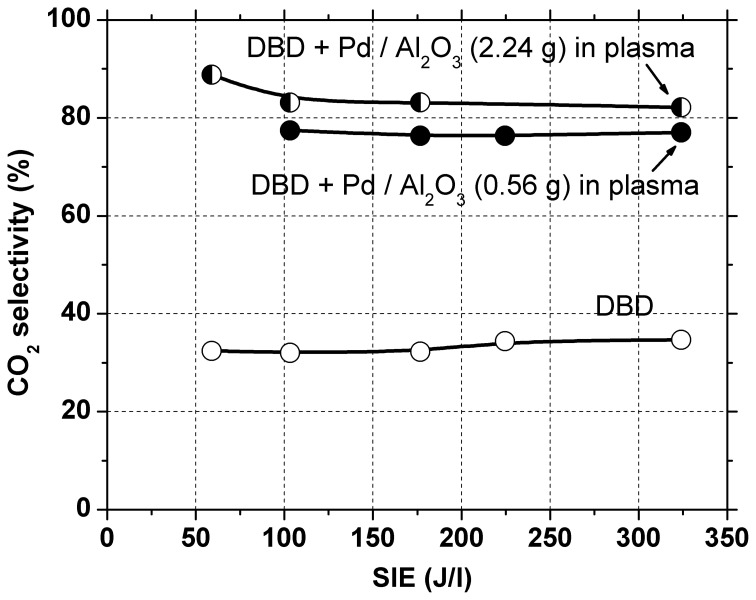
**CO_2_ selectivity as a function of SIE in the plasma and in the plasma–catalytic system**.

In a first set of experiments 2.24 g catalyst were introduced in the DBD reactor, occupying the entire discharge gap. Initially, the gas was passed through the reactor for several hours with plasma off, so that toluene was adsorbed on the catalyst bed until saturation was reached. After the plasma was turned on, a large amount of toluene was desorbed from the catalyst. Therefore, a much higher toluene concentration is actually present in the plasma-catalytic reactor as compared to the input concentration. Since it is well known that conversion decreases with increasing concentration, poorer toluene conversion as compared to that obtained in the DBD is expected. Indeed, toluene conversion is considerably lower than the above-mentioned values, remaining below 10% even at the highest SIE used. Although an important part of the toluene was converted mainly into CO_2_ (selectivity 80–90%, see Figure 3), the effluent gas still contained a high concentration of toluene, therefore this configuration was not effective for total toluene removal.

In the following experiments only 0.56 g of catalyst were placed in the discharge region. In this case the toluene conversion was similar with that obtained with plasma alone. The amount of toluene removed is actually higher, because part of the toluene adsorbed on the catalyst is also oxidized. This is evidenced also by the concentration of carbon oxides (CO + CO_2_) in the effluent gas, which increased significantly (with 40–70%) as compared to the results obtained with plasma alone, due to conversion of adsorbed toluene. The selectivity to CO_2_ was 76–77% regardless of SIE. Clearly, the presence of Pd/Al_2_O_3_ catalysts significantly improved the process selectivity toward total oxidation.

**Figure 4:**
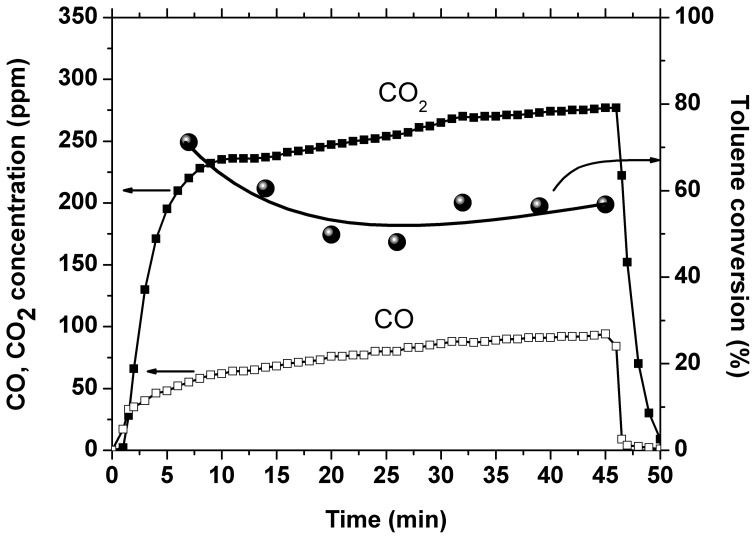
**Temporal evolution of CO and CO_2_ concentrations and toluene conversion**.

Another experiment was performed with 0.56 g catalyst placed inside the discharge area and 1.7 g catalyst placed downstream of the plasma reactor. This experiment was carried out for an amplitude of the applied voltage of 17 kV, corresponding to a SIE of 225 J/L. The temporal evolution of toluene conversion and CO_2_ and CO concentrations is shown in Figure 4. The conversion showed an initial decrease of about 15% and then became stable around 56%, which represents an improvement as compared to the result obtained with plasma alone: 41% at the same SIE. The CO_2_ selectivity showed also a slight decrease in time, from 80 to 75%, but is still largely enhanced as compared to 35% in plasma. Initially the carbon balance is very close to 100%, while after several minutes of plasma operation the concentration of carbon oxides exceeds considerably the theoretical value. This behavior, together with the conversion decrease are most likely due also to the desorption of toluene from the catalyst. Even after the conversion stabilizes, the concentration of COx is almost two times higher than the maximum possible value, which suggests that the amount of oxidized toluene is actually higher than the 50 ppm introduced. Therefore, plasma and ozone generated in the DBD have the ability to oxidize toluene on the catalyst surface.

**Figure 5:**
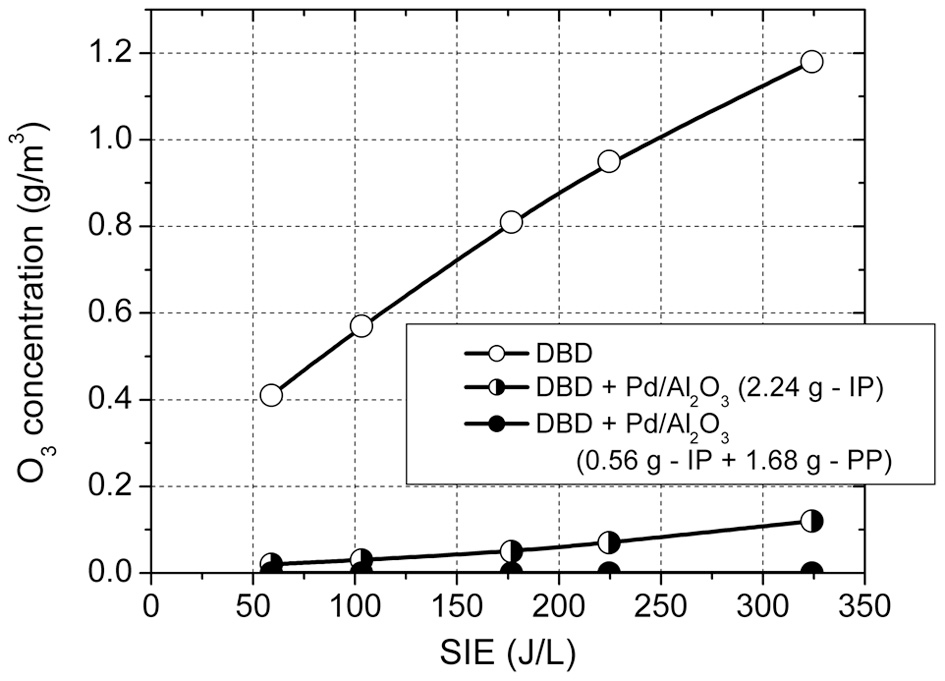
**Concentration of ozone in DBD and in the plasma catalytic system**.

A further advantage of using this configuration is the complete removal of ozone in the effluent gas. Figure 5 shows the ozone concentration generated in the DBD in the absence of catalysts, with the plasma reactor fully packed with catalyst and with 0.56 g catalyst placed in plasma and the rest (1.68 g) placed post-plasma. In plasma, in the absence of catalysts, the O_3_ concentration increased with increasing SIE in the range 0.4–1.18 g/m^3^. The O_3_ concentration was reduced to 0.02–0.12 g/m^3^ with 2.24 g of catalyst introduced in the plasma zone and was completely removed when the catalyst was placed in the combined in-plasma–post-plasma configuration. It is therefore obvious that ozone generated in the plasma plays an important role in toluene oxidation. The most likely mechanism is ozone decomposition on the catalyst surface, forming highly reactive atomic oxygen, which reacts with toluene and also shifts the product distribution toward total oxidation.

### Catalysts characterization

**Figure 6:**
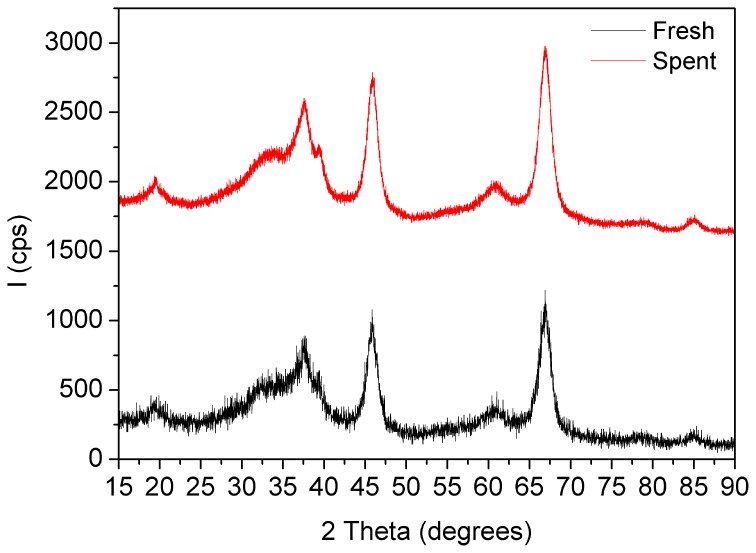
**XRD patterns of the fresh and spent Pd/γ-Al_2_O_3_ catalyst**. Spent catalyst was collected from inside the plasma reactor.

**Figure 7:**
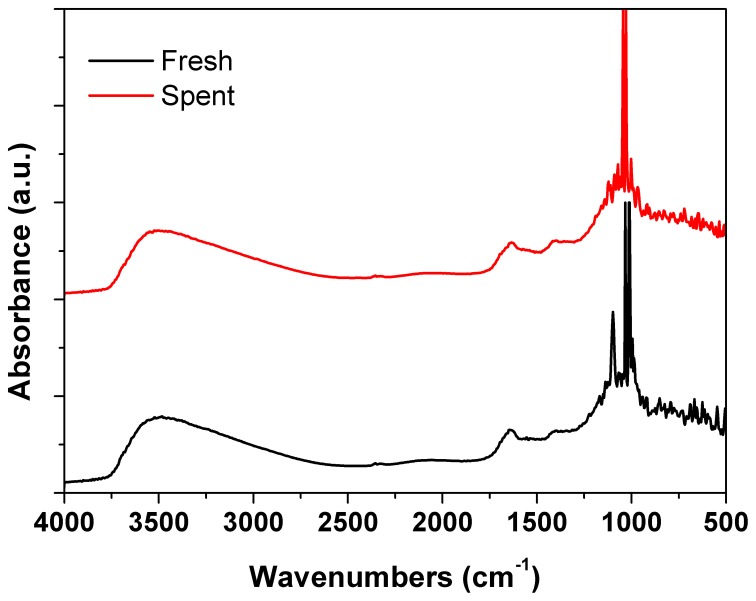
**DRIFTs spectra of the fresh and spent Pd/γ-Al_2_O_3_ catalyst**. Spent catalyst was collected from inside the plasma reactor.

**Figure 8:**
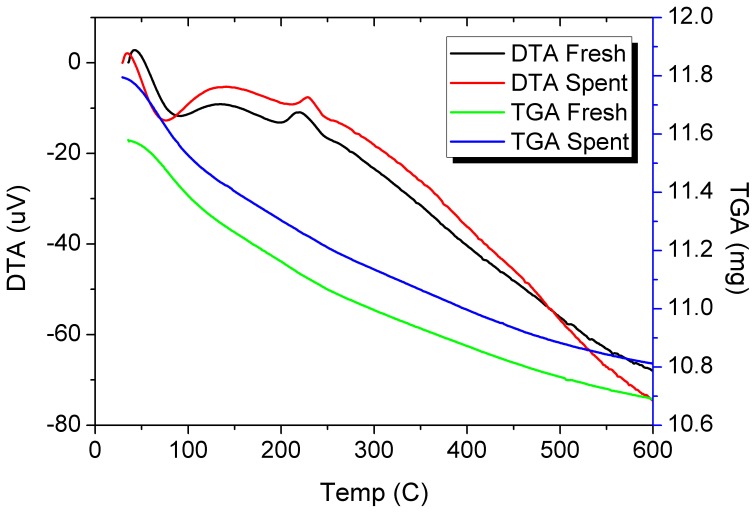
**TGA and DTA profiles of the fresh and spent Pd/γ-Al_2_O_3_ catalyst**. Spent catalyst was collected from inside the plasma reactor.

**Figure 9:**
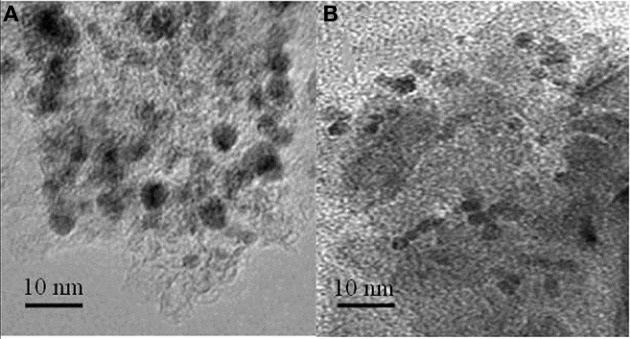
**TEM pictures of the Pd/γ-Al_2_O_3_ catalyst: before (A), and after (B) the plasma/catalytic test**. Spent catalyst was collected from inside the plasma reactor.

Figures 6–9 present results using spent catalysts collected from inside the plasma reactor. However, similar characterizations were obtained with catalysts inside the plasma, downstream of plasma reactor and both inside and after the reactor.

X-Ray diffractograms of the fresh and spent catalysts showed only diffraction planes of γ-Al_2_O_3_ with reflections located at 2Θ 19.4° (111), 32.5° (220), 37.48° (011), 39.3° (222), 45.8° (400), 60.7 (511), 66.9° (440), and 84.8° (444) respectively (PDF Card 00-050-0741) (Figure 6). None of them exhibited diffraction lines associated with palladium that accounts firstly for the good dispersion of the metal, with a particle size under the detection limit of XRD technique, and secondly for the fact that working under plasma irradiation for more than 24 h induced no agglomeration.

DRIFTs spectra provided additional arguments concerning the stability of the catalyst under the reaction conditions (Figure 7). The strong broadening at 3800–3000 cm^−1,^ with a maximum around 3500 cm^−1^ occurs due the OH groups bending vibration. This band is accompanied by a band at about 1640 cm^−1^ which is assigned to the stretching vibration in the absorbed and coordinated water. The stronger broadening band at 1000–500 cm^−1^ corresponds to Al-O vibration. The band at ~1092 cm^−1^ is assigned to Al-OH symmetric bending, the band at ~1170 cm^−1^ is assigned to Al-OH asymmetric bending and the band at ~1396 cm^−1^ is assigned to Al-OH stretching. Working under plasma did not affected the presence of the OH groups, but only induced a decrease of the intensity of these bands that was caused by the deposition of a small amounts of carbonaceous deposits. Thermal analysis was not able to clearly detect the presence of these deposits neither from the TGA nor from the DTA profiles (Figure 8). However, elemental analysis of the fresh and spent samples identified, indeed, small amounts of carbon on the spent catalyst (Table 1). While it showed no difference in the nitrogen and sulfur content, a very slight increase in the carbon content was detected that is typical for radicalic processes where a polycondensation of the aromatic ring can occur.

**Table 1:** **Elemental analysis of fresh and spent catalyst**.

**Sample**	**Content, wt%**			
	**N**	**C**	**H**	**S**
Fresh Pd/γ-Al_2_O_3_ catalyst	0.048	0.339	0.886	–
Spent Pd/γ-Al_2_O_3_ catalyst	0.050	0.539	0.888	–

TEM pictures of the fresh and spent catalyst also reveal that the exposure to the reaction conditions described above did not generate changes in the morphology of palladium (Figure 9). Fresh catalysts exposed Pd particles with sizes in the range 4.7–10 nm, and an average size of 6.5 nm (Figure 9A). Spent catalysts exposed Pd particles with sizes in a narrower range 3.8–7.1 nm, and an average size of 5.1 nm (Figure 9B). Accordingly these measurements suggest only a redispersion of the large particles.

## Discussion

The efficiency of a plasma-catalyst hybrid system has already been demonstrated by other studies (Magureanu et al., 2005, 2006, 2007) and the present results provide additional confirmation of such a behavior. However, up to date no clear evidence of the stability of the catalyst has been provided in the literature. Our studies demonstrate that the investigated catalyst was not affected by the plasma conditions or by a deposition of soot. Except a very small change in the intensity of the OH bonds belonging to the support no other changes have been evidenced. These results are well correlated to the catalysts characterization. The presence of small palladium particles limited a redispersion of the metal during the plasma-catalytic oxidation of toluene and conserved the catalytic behavior with a good stability.

Numerous studies have shown that the formation of free radicals or ion-radicals is the first and decisive stage for the non-oxidative conversion of hydrocarbons in non-equilibrium plasmas (Schmidt-Szałowski et al., 2011). Obviously, the radicalic pathway, in addition to the oxidation of the hydrocarbon, is also matter of polymerization of small derived entities with the formation of large polyaromatic structures, namely, soot. The extremely small amount of deposited carbon (see Table 1) demonstrated that for the concentration of VOC and SIE used in this study the cooperative plasma/catalytic oxidation of the toluene and derived entities occurred to a large extent.

## Concluding remarks

The results collected in this study confirmed the efficiency of Pd/γ-Al_2_O_3_, not only as an active oxidation catalyst for the combustion of the hydrocarbons, but also as an efficient catalyst in a hybrid configuration with non-thermal plasma. In addition, the characterization results using elemental and thermal analysis, XRD, and DRIFT provided evidence about the stability of this catalyst under the investigated conditions. Noteworthy, these characterizations evidenced the formation of soot only in a very small amount that is also in agreement with previous reports (Holzer et al., 2002).

### Conflict of interest statement

The authors declare that the research was conducted in the absence of any commercial or financial relationships that could be construed as a potential conflict of interest.
